# Methods of Cleaning Taps to Prevent Hospital-Associated Infections: An Environmental Survey-Based Study

**DOI:** 10.3390/idr15010015

**Published:** 2023-02-20

**Authors:** Masayoshi Hashimoto, Satomi Asai, Kazuo Umezawa, Ryosuke Tanitsu, Miki Miyazawa, Michiko Kobayashi, Yuji Kawakami, Yoshika Sekine, Yuji Suzuki, Hayato Miyachi, Kenji Okami

**Affiliations:** 1 Department of Pharmacy, Tokai University Hospital, Isehara 259-1193, Japan; 2 Division of Infection Control, Tokai University Hospital, Isehara 259-1193, Japan; 3 Department of Laboratory Medicine, Tokai University School of Medicine, Isehara 259-1193, Japan; 4 Department of Emergency Medicine and Critical Care, Tokai University School of Medicine, Isehara 259-1193, Japan; 5 Department of Nursing, Tokai University Hospital, Isehara 259-1193, Japan; 6 Department of Environmental Science and Education, Tokyo Kasei University, Tokyo 173-8602, Japan; 7 Department of Chemistry, School of Science, Tokai University, Hiratsuka 151-8677, Japan; 8 Department of Otolaryngology-Head and Neck Surgery, Tokai University School of Medicine, Isehara 259-1193, Japan

**Keywords:** infection control, water supply, carbapenem-resistant *Enterobacteriaceae*, disinfectants, health facility environment

## Abstract

In hospitals, outbreaks can occur due to pathogens accumulating in the areas around the wards’ washbasins. Carbapenem-resistant Enterobacterales (CRE) was detected in an environmental survey in the high-care unit of a university hospital in Isehara, Japan, and effective cleaning methods were investigated. This study investigated methods of cleaning taps using commonly used detergents and disinfectants, and it assessed their effectiveness in removing hard scale and pathogens, including CRE. The taps were cleaned using various methods and cleaning agents, including environmentally neutral detergent, citric acid, baking soda, cleanser, 80% ethanol, 0.1% sodium hypochlorite, and a phosphoric acid-based environmental detergent (Space Shot). The cleaning effect was assessed based on the agent’s effectiveness at removing hard scale from taps. Biofilms and scale were identified on taps, and several bacterial species were cultured. Only phosphoric acid-based detergent was effective at removing hard scale. After cleaning with the phosphoric acid-based detergent, the bacterial count decreased, and no CRE or other pathogens were detected. These results provide a reference for other facilities considering introducing this cleaning method.

## 1. Introduction

The areas around washbasins in hospital wards used by staff and patients are contaminated by pathogenic bacteria [1,2]. An outbreak of drug-resistant *Acinetobacter baumannii* occurred in the intensive care unit of our Advanced Emergency and Critical Care Center of Tokai University Hospital due to a biofilm of drug-resistant *Acinetobacter baumannii,* which adhered to the inside of a washbasin tap and contaminated the water supply pipe for a prolonged period [3]. Immunosuppressed patients are prone to develop opportunistic infections caused by environmental bacteria [4]; therefore, it is important to keep washbasins, including taps, clean in hospital wards. 

We conducted an environmental survey of washbasin units, including the taps, in the high-care unit (HCU, 36 beds) of the Advanced Emergency and Critical Care Center of Tokai University Hospital in 2018. We detected the presence of *Enterobacter cloacae*, a carbapenem-resistant Enterobacterales (CRE), in a tap. CRE is highly drug resistant, and CRE infections lead to longer hospital stays and higher medical costs than non-CRE infections [5]. The treatment and cure of pneumonia as well as bloodstream infections caused by CRE can be difficult owing to the limited availability of effective antibiotics, and the reported mortality is high [6]. Furthermore, CRE has the propensity to spread via plasmids, thus posing the risk of causing nosocomial outbreaks [7]. Therefore, the prevention of horizontal transmission of CRE is crucial for infection control.

Biofilms and scales that adhere to taps are difficult to clean because they harbor numerous bacteria [8,9], and some biofilms and scales contain CRE. Methods for cleaning taps effectively and efficiently remain unclear. This study aimed to investigate effective methods for cleaning and disinfecting taps using commonly used detergents and disinfectants by conducting a bacteriological survey for areas around washbasins, including taps.

## 2. Materials and Methods

**Figure 1:**
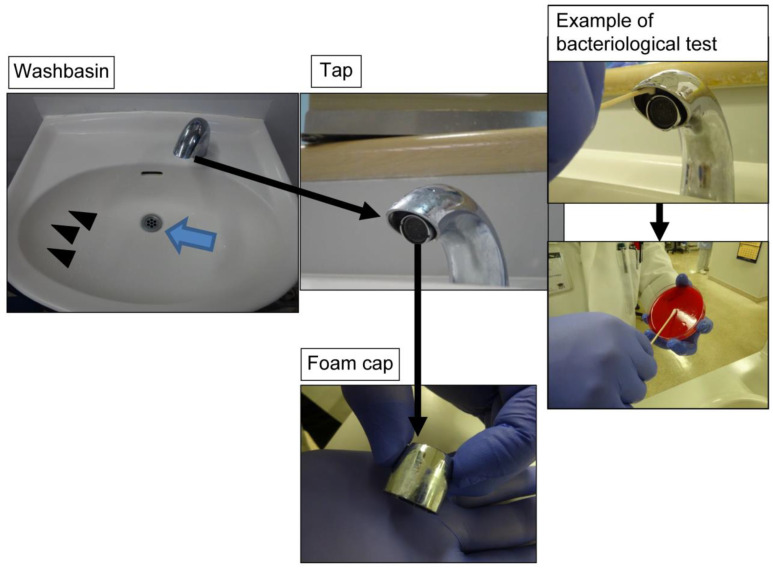
Bacteriological survey of washbasin units. We conducted a bacteriological survey of the drain (blue arrow), the sides of the sink (arrowheads), and the tap of washbasins in the high-care unit of the Advanced Emergency and Critical Care Center. The taps had a foam cap on the inside. Samples were collected from the tap for bacteriological testing by simultaneously wiping the inner foam cap and the inside surface of the tap with a sterile cotton swab.

We conducted a bacteriological survey around four washbasins in the HCU shared by patients for brushing their teeth and gargling (Figure 1). Swab samples were collected from drains, sink sides, and taps, including the inside surface of the tap and foam caps, using one sterilised cotton Nissui swab (Nissui Pharmaceutical Co., Ltd., Tokyo, Japan) per environmental surface. After sample collection, the swabs were immediately applied to blood agar, cultured in an incubator at 37 °C for 24 h, and then allowed to stand at room temperature for 24 h. The bacterial count was measured according to a semi-quantitative culture method [10,11], and the number of bacteria on culture was quantified according to the *Clinical Microbiology Procedures Handbook* guidelines as follows: colony growth on less than 1/3 of the medium = 1+; 1/3 to less than 2/3 = 2+; 2/3 or more = 3+; and the entire medium = 4+ [12]. Bacterial identification and susceptibility testing of the isolates were performed to obtain minimum inhibitory concentration (MIC) values using a microdilution method (CLSI, 2018) and a DxM Microscan WalkAway (Beckman Coulter Inc., Brea, CA, USA) microorganism identification susceptibility analyser. Genotyping was conducted to identify drug-resistant bacteria, as described previously [13].

Based on the results of the bacteriological survey, we focused on evaluating different methods for cleaning taps using various cleaning agents, including an environmentally neutral detergent (Mypet, Kao Corporation, Tokyo, Japan), citric acid, baking soda, a cleanser (Kaneyo Soap Co., Ltd., Fukui, Japan), 80% ethanol, 0.1% sodium hypochlorite, and an environmental detergent (Space Shot rust remover/toilet cleaner, Orb Tech Co., Ltd., Tokyo, Japan), hereinafter referred to as Space Shot. Space Shot, a strongly acidic phosphoric acid-based environmental cleaning agent, is composed of phosphoric acid, ethanol, hydrochloric acid, zinc, dye, and water. Space Shot has a pH of approximately 1.6, which is lower than that of citric acid. The cleaning effect was evaluated based mainly on its effect on removing hard scale from taps.

## 3. Results

**Figure 2:**
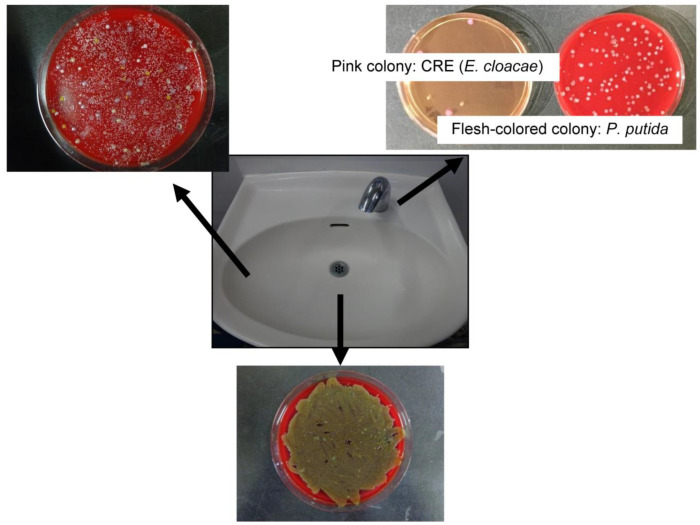
Summary of bacteriological survey results. The bacteriological survey revealed a wide variety of bacteria in four washbasins in the high-care unit. Many bacteria were detected in the drains and on the sides of the sinks. *Pseudomonas putida* (pink colonies, top right) and CRE (flesh-coloured colonies, top right), which are pathogenic bacteria, were detected on washbasin taps. CRE, carbapenem-resistant Enterobacterales.

The taps of the washbasins in hospital rooms were extremely contaminated (Figure 2). In the bacteriological survey of the environment, many bacteria were cultured from washbasin drains. The bacterial count was high on the sides of sinks and taps (including the inside surface of the tap and the outside surface of the foam cup). The following pathogenic bacteria were identified: *Micrococcus* sp., *Bacillus* sp., coagulase-negative *Staphylococcus*, *Staphylococcus aureus*, gram-positive rods, *Stenotrophomonas maltophilia*, *Corynebacterium* sp., glucose non-fermentative gram-negative bacilli, *Enterobacter cloacae*, *Exophiala* sp., *Candida* sp., *Pantoea agglomerans*, *Pseudomonas* sp., *Citrobacter braakii*, *Pseudomonas aeruginosa*, alpha-hemolytic *Streptococcus*, *Acremonium* sp., *Aspergillus niger*, *Fusarium* sp., *Rhizobium radiobacter*, *Weeksella virosa*, *Acinetobacter lwoffii* group, and *Cladosporium* sp. Bacteria detected on taps included pathogenic *Enterobacter cloacae* and *Pseudomonas putida* (Table 1). These were detected in the drain, on the sides of the basin, and on the tap. The *Enterobacter cloacae* isolate harbored *AmpC* and *CTX-M* resistance genes.

**Figure 3:**
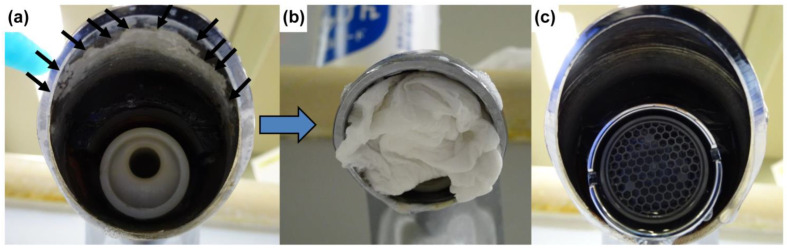
State of a tap before and after cleaning. The foam cap was removed. (**a**) A large amount of limescale is attached to the tap (black arrows). (**b**) The inside of the tap was washed after applying a gauze that was soaked with Space Shot, a phosphoric acid-based industrial detergent, for 30 min. During cleaning, the foam cap was taken out and cleaned. (**c**) After cleaning, the tap did not have any visible hard scale and appeared to be clean. The bacteriological survey that was conducted immediately afterward also detected very few bacteria.

The hard scale on the taps could not be removed by physically scraping it off with a clip or screwdriver nor by soaking tissue paper with a chemical solution, applying it to the scale, and leaving it for over 30 min. Cleaning the taps with an environmentally neutral detergent, citric acid, baking soda, a cleanser, 80% ethanol, or 0.1% sodium hypochlorite also had little impact on the scale on both the inner surface of the tap and the foam cap (Table 2). Application of tissue paper soaked with Space Shot for 30 min resulted in a softening of the hard scale and allowed easy removal of the scale without using physical force. We visually confirmed that the tap was clean (Figure 3), and a bacteriological survey cultured a few bacteria (Table 3). No CRE or other pathogens were detected. Space Shot was the only cleaning agent that was effective in removing hard scale. Despite its strong detergent effect, Space Shot did not result in corrosion or discoloration of the taps.

## 4. Discussion

The hard scale adhering to taps consists mainly of alkaline deposits such as calcium carbonate. According to our findings, Space Shot was the only agent tested that could effectively remove hard scale. This is probably because the low pH of this product acts on alkaline deposits.

According to a survey by the Ministry of Land, Infrastructure, Transport and Tourism in Japan, there are only nine countries where tap water is drinkable [14]. In Japan, tap water is hygienic and is considered potable [15]. Hospitalised, ambulatory patients wash their hands, brush their teeth, and gargle at the washbasins. Therefore, the detection of even a small number of pathogenic microorganisms in tap water used by patients, such as in high-care units where immunocompromised patients are hospitalised, poses an infection risk. Outbreaks have been reported due to the contamination of tap and drinking water with various microorganisms [3,16,17,18,19,20,21]. Notably, a previous report suggests that more than half of the bacteria detected in tap water showed antimicrobial resistance [22], as did our findings. Hand and equipment contamination from the taps and surrounding sink surfaces can promote transmission of antimicrobial-resistant pathogenic bacteria, which may result in hospital-associated infections. In a study investigating the genotype of *P. aeruginosa* contaminating the tap of a surgical intensive care ward, 5 of 17 cases (29%) had the same genotype as *P. aeruginosa* detected in the tap over a 7-month period [21]. This suggests that the tap serves as an environmental reservoir of pathogenic microorganisms. It is clear that it is important to remove bacteria adhering to taps to prevent infections caused by pathogens in the environment. In this study, we immediately implemented cleaning after CRE was detected in the environmental survey. The Advanced Emergency and Critical Care Center actively conducts bacteriological tests on patients on hospital admission and discharge; however, to date, no CRE has been detected on discharge in patients who used the taps in the centre. CRE may have adhered to taps due to splash-back from the washbasin during hand washing and gargling by patients with CRE colonisation. When drug-resistant bacteria, such as CRE, adhere to a tap, it is possible that patients who use water from the tap contract CRE infection. No pathogenic bacteria, including CRE, were detected in patients who used the washbasins after effective cleaning of the taps, suggesting that effective cleaning can prevent horizontal infection from the environment.

In an outbreak of CRE detected in the water supply of an intensive care unit (ICU), it was reported that even deep cleansing with sodium hypochlorite and pressurised steam decontamination at 170 °C could not remove CRE [23]. A strength of our study is that we found an effective and efficient cleaning method for removing CRE. Introducing this method may be useful in other medical facilities with CRE in the water supply.

Our study has some limitations. First, the bacterial count values were not determined and a quantitative comparison was not possible. Second, the investigation of various detergents did not include a bacteriological investigation and the cleaning effect was judged only with the naked eye. Third, we did not determine the optimal interval at which Space Shot should be used for disinfection. Further research is required to address these questions.

To maintain hygiene, sinks should be cleaned daily, and taps should be cleaned regularly. The appropriate interval for cleaning taps is currently unclear. At our facility, due to human resource and cost issues, taps are cleaned once every 6 months using Space Shot. During the past 4 years since the implementation of this cleaning method, we have not detected new hard scale build-up or associated pathogenic bacteria on taps and have not detected any hospital-associated infections attributable to tap water contamination. Establishing an effective method of cleaning taps as part of hygiene management is important for hospital infection control.

## 5. Conclusions

Taps can serve as an environmental reservoir for pathogenic microorganisms, such as CRE. A phosphoric acid-based detergent (Space Shot) effectively removed hard scale from taps and washbasins. Effective cleaning of the taps as part of the hygiene management of washbasins is important for hospital infection control.

## Figures and Tables

**Table 1:** The antimicrobial susceptibility pattern of the isolates from washbasin taps in medical facilities.

Species	β-Lactams								AGs		FQs	Other Agents	
	IPM	MEPM	PIPC	CAZ	CFPM	CTRX	TAZ/PIPC	AZT	GM	AMK	LVFX	MINO	ST
*Enterobacter cloacae*	>2 (R)	>2 (R)	>64 (R)	>8 (R)	>16 (R)	>2 (R)	64 (I)	≤4 (S)	≤2 (S)	≤4 (S)	≤0.5 (S)	>8 (R)	≤2/38 (S)
*Pseudomonas putida*	≤1 (S)	4 (S)	≤8 (S)	≤4 (S)	≤2 (S)	NT	≤8 (S)	8 (S)	≤2 (S)	≤8 (S)	0.5 (S)	≤2 (S)	>2/38 (R)

AGs, aminoglycosides; AMK, amikacin; AZT, aztreonam; CAZ, ceftazidime; CFPM, cefepime; CTRX, ceftriaxone; FQs, fluoroquinolone; GM, gentamicin; IPM, imipenem; LVFX, levofloxacin; MEPM, meropenem; MIC, minimum inhibitory concentration; MINO, minocycline; NT, not tested; PIPC, piperacillin; TAZ/PIPC, tazobactam/piperacillin: R, resistant; S, susceptible; ST, sulfamethoxazole/trimethoprim.

**Table 2:** Effectiveness of various detergents at removing hard scale around the washbasin tap.

Detergent	Adhesion of Scale after Cleaning
Mypet	Large amount (no change)
Citric acid	Large amount (no change)
Baking soda	Large amount (no change)
Cleanser	Large amount (no change)
80% ethanol	Large amount (no change)
0.1% sodium hypochlorite	Large amount (no change)
Space shot	No visible scale

**Table 3:** Scale and bacteria detection status before and after cleaning around the washbasin tap with Space Shot detergent.

Cleaning Status	Appearance of the Scale	Bacterial Species	Amount of Bacteria ^a^
Before	Large amount	*Exophiala* sp.	1+
		*Cladosporium* sp.	1+
		Coagulase-negative *Staphylococcus*	1+
After	None	*Cladosporium* sp.	1 colony

^a^ The number of bacteria on culture was quantified according to the *Clinical Microbiology Procedures Handbook* guidelines [12]. 1+ indicates colony growth on less than 1/3 of the medium.

## Data Availability

The paper provides all the relevant data supporting the results.
